# Sulfate Transporters in Dissimilatory Sulfate Reducing Microorganisms: A Comparative Genomics Analysis

**DOI:** 10.3389/fmicb.2018.00309

**Published:** 2018-03-02

**Authors:** Angeliki Marietou, Hans Røy, Bo B. Jørgensen, Kasper U. Kjeldsen

**Affiliations:** Center for Geomicrobiology, Department of Bioscience, Aarhus University, Aarhus, Denmark

**Keywords:** sulfate transporter, sulfate-reducing microorganisms, SulP, CysP, CysZ, DASS

## Abstract

The first step in the sulfate reduction pathway is the transport of sulfate across the cell membrane. This uptake has a major effect on sulfate reduction rates. Much of the information available on sulfate transport was obtained by studies on assimilatory sulfate reduction, where sulfate transporters were identified among several types of protein families. Despite our growing knowledge on the physiology of dissimilatory sulfate-reducing microorganisms (SRM) there are no studies identifying the proteins involved in sulfate uptake in members of this ecologically important group of anaerobes. We surveyed the complete genomes of 44 sulfate-reducing bacteria and archaea across six phyla and identified putative sulfate transporter encoding genes from four out of the five surveyed protein families based on homology. We did not find evidence that ABC-type transporters (SulT) are involved in the uptake of sulfate in SRM. We speculate that members of the CysP sulfate transporters could play a key role in the uptake of sulfate in thermophilic SRM. Putative CysZ-type sulfate transporters were present in all genomes examined suggesting that this overlooked group of sulfate transporters might play a role in sulfate transport in dissimilatory sulfate reducers alongside SulP. Our *in silico* analysis highlights several targets for further molecular studies in order to understand this key step in the metabolism of SRMs.

## Introduction

Dissimilatory sulfate reduction is a key metabolic process in anoxic environments. It is catalyzed by sulfate-reducing microorganisms (SRM), which encompass a diverse group of bacteria and archaea spanning several phylogenetic lineages (Müller et al., 2015). SRM use sulfate as terminal electron acceptor for the oxidation of organic electron donors or hydrogen, thereby producing sulfide, which they release back to the environment (dissimilatory sulfate reduction). Sulfate can also be reduced and assimilated for the synthesis of cell material whereby the produced sulfide is incorporated into sulfur-containing compounds like cysteine (assimilatory sulfate reduction). The initial two steps of sulfate metabolism are similar in both dissimilatory and assimilatory pathways of sulfate reduction. The sulfate ion is first transported through the hydrophobic membrane into the cytoplasm and then activated with ATP forming the intermediate APS (adenosine-5-phosphosulfate). Since the sulfate ion cannot enter the cell by passive diffusion, a specialized transport system is required by all sulfate-utilizing microorganisms.

Despite the growing knowledge on the physiology of dissimilatory sulfate reduction most of the information regarding the sulfate transport proteins involved in the dissimilatory pathway has been adopted from the assimilatory pathway literature. There are five major families of sulfate transporter proteins that have been previously identified in assimilatory sulfate-reducing prokaryotic and eukaryotic organisms (Kertesz, 2001; Piłsyk and Paszewski, 2009; Aguilar-Barajas et al., 2011): (1) The SulT sulfate transporters [Transporter Classification DataBase (TCDB) 3.A.1.6] are members of the ATP-Binding Cassette (ABC) (TCDB 3.A.1) superfamily of proteins that transport a wide range of molecules but also participate in several cellular processes and have been extensively characterized in *Escherichia coli* and *Salmonella* sp. (Dassa and Bouige, 2001; Saier et al., 2006). (2) The SulP sulfate transporters (TCDB 2.A.53) which are part of the Amino acid-Polyamine-organoCation (APC) superfamily of proteins (TCDB 2.A.3) that function as solute:cation and solute:solute antiporters and have been characterized in *Mycobacterium tuberculosis* (Saier, 2000; Zolotarev et al., 2008). (3) The CysP sulfate transporters (TCDB 2.A.20.4.1), which are members of the inorganic Phosphate Transporter (TCDB 2.A.20) family (PiT) that function mainly as inorganic phosphate or sulfate transporters with either H^+^ or Na^+^ symport and have been characterized in *Bacillus subtilis* (Saier et al., 1999; Mansilla and de Mendoza, 2000). (4) The DASS (Divalent Anion: Na^+^ Symporter) sulfate transporters (TCDB 2.A.47) which belong to the Ion Transporter (IT) superfamily of secondary carriers of cationic and anionic compounds have been characterized in *Rhodobacter capsulatum* (Prakash et al., 2003; Gisin et al., 2010). (5) The CysZ sulfate transporters, members of the TSUP (Toluene Sulfonate Uptake Permease) family (TCDB: 2.A.102). The TSUP transporters form a ubiquitous and diverse family of transmembrane proteins that are poorly characterized but predicted to be involved in the transport of sulfur-based molecules (Shlykov et al., 2012).

Sulfate concentrations vary in the environment from 28 mM in seawater to a few micromolar in freshwater environments and in the gastrointestinal tract of animals. Microorganisms have developed dual kinetic approaches to transport sulfate efficiently at varying concentrations. Concurrent high and low affinity sulfate reduction was reported in marine sediments (Tarpgaard et al., 2011). At high sulfate concentrations, sulfate is transported into the cell using an electroneutral system driven by the pH gradient across the membrane, where two cations are symported per sulfate (Cypionka, 1989). When the sulfate concentration in the environment is extremely low, an electrogenic sulfate transport system is used that symports three cations per sulfate molecule (Cypionka, 1989). The steady state accumulation of sulfate in symport with H^+^ or Na^+^ can be described by the equation:

(1) $$\text{Log}({\text{c}}_{\text{i}}/{\text{c}}_{\text{o}})=-(\text{m}+\text{n})\text{ΔΨ}/\text{Z}+\text{n}\cdot \text{ΔpH}$$

where c_i_ and c_o_ are the sulfate concentrations inside and outside of the cell, respectively; m is the charge of sulfate; n is the number of symported cations; ΔΨ is the electrical potential, Z is equal to 2.3.R.T/F which is about 60 mV at 30°C; ΔpH is the pH gradient across the membrane (Cypionka, 1989, 1995). At neutral external pH the average ΔpH is 0.5 and if we assume the electrical potential to be -150 mV, then electroneutral symport with two cations would result in 10-fold accumulation (low affinity transport), while electrogenic symport with three cations would allow 10,000-fold accumulation of sulfate (high affinity transport) (Cypionka, 1989). A 20,000-fold accumulation was reported at external sulfate concentrations of 0.1 μM in *Desulfovibrio salexigens* (Kreke and Cypionka, 1994).

High accumulation of sulfate was only observed under sulfate-limitation (less than 1 mM of sulfate) suggesting regulation of the high affinity transport system at the molecular level (Stahlmann et al., 1991). A low affinity system operates when sulfate concentration is 1 mM or higher; the high affinity system is then switched off in order to save energy and prevent an over-accumulation of sulfate inside the cell (Stahlmann et al., 1991). The marine sulfate reducer *Desulfobacterium autotrophicum* switched from low affinity to high affinity sulfate reduction when the concentration of sulfate dropped below 500 μM with a concomitant change in the expression of genes encoding putative sulfate transport proteins from the SulP and DASS families (Tarpgaard et al., 2017).

With the help of comparative genomics this review aimed to (i) identify putative homologs of sulfate transporters in SRM, (ii) determine their taxonomic distribution and their genomic context and hereby infer their putative function as sulfate transporters, and (iii) provide a reference roadmap of putative sulfate transporters in SRM for further explorations.

## Analytical Approach

### Protein Identification

The IMG/MER online database (Markowitz et al., 2011) was searched (accessed on 15 December 2015) using the following PFAM models for transporter families with members known to transport sulfate across the cytoplasmic membrane using the default search parameters of the Function Search option: PFAM00916 (SulP family), PFAM00939 (DASS family), PFAM01384 (CysP family). Since there was no PFAM model available for the CysZ-family of transporters the transporter classification number TC9.A.29 for the TSUP protein family was used instead for the Function Search option at the IMG/MER database (Saier et al., 2013). For the SulT family of transporters the IMG/MER database was searched using TIGRFAM models (TIGR00968, TIGR00969, and TIGR00971) and blastp (*E*-value cut-off: 1e-5) using the SulT family sulfate permease protein sequence from *Salmonella enterica* LT2 (AAL22903) as query. The genomes selected for each search query were at the “Finished” status in the Bacteria and Archaea domains to avoid false negative results due to incomplete genome sequencing. To confirm that the genomes selected for this study belonged to dissimilatory SRM the literature, DSMZ strain records, and NCBI submission information files were reviewed. Transmembrane helix prediction was performed using the TMHMM Server v. 2.0 (Krogh et al., 2001).

### Phylogenetic Analysis

The pairwise percent amino acid sequence similarity between each putative sulfate transporter identified and the model sulfate transporter sequence of a given family was calculated using the EMBOSS Needle tool provided by the EMBL-EBI webservice (McWilliam et al., 2013). The model protein sequences for each sulfate transporter protein family were CysP from *B. subtilis* (BSUA_01689), CysZ from *Corynebacterium glutamicum* (CAF20834), DASS from *Rhodobacter capsulatus* (WP_013066626), and SulP from *M. tuberculosis* (Rv1739c).

For the phylogenetic analysis of the putative sulfate transporters multiple sequence alignments of each protein family were constructed using the MAFFT program provided by the EMBL-EBI webservice with default settings (McWilliam et al., 2013; Katoh et al., 2017). The alignments were imported into ARB where alignment columns were selected for phylogenetic analyses using a 30% conservation filter (Ludwig et al., 2004). The resultant alignments including only the more conserved positions were used for constructing maximum likelihood phylogenetic trees in RAxML v8.2.9 (Stamatakis, 2014). Trees were inferred under the PROTGAMMA-LG model with empirically determined amino acid frequencies. The LG substitution model was initially empirically determined to best fit the datasets using the PROTGAMMAAUTO tool in RAxML. The phylogenetic trees were visualized using iTOL (Letunic and Bork, 2006). Multiple sequence alignments were viewed and edited by the Geneious R7 software (Kearse et al., 2012).

### Genomic Context

The gene locus tag number was used in order to calculate the number of genes separating the putative sulfate transporter genes from genes of the canonical sulfate reduction pathway (*sat*, *apr*A, *dsr*A, *dsrC*) and the associated membrane-bound electron transport complexes QMO (*qmo*C) and DsrMKJOP (*dsrM)* (Pereira et al., 2011). The genomic context analysis was performed initially for all the putative sulfate transporters identified by our searches. We also performed the genomic context analysis using a subset of the putative sulfate transporters, which according to the phylogenetic analysis clustered with the model sequence (functionally characterized sulfate tranporter) for each protein family with at least 50% bootstrap clade support. Proteins in this subset were considered as more likely to function as sulfate transporters and we therefore examined their genomic context separately.

## Sulfate Transporters in Dissimilatory Sulfate Reducing Bacteria and Archaea

We surveyed the closed genomes of 38 taxonomically diverse sulfate-reducing bacteria and 6 sulfate-reducing archaea known to be able to carry out dissimilatory sulfate reduction for the presence of the five different sulfate transporter protein families (**Table 1**). We were not able to detect any SulT family members using either the TIGRFAM models or by BLAST (see Analytical Approach section) despite the prevalence of several ABC-type transporters in the genomes of the SRM that were annotated as sulfate transporters.

**Table 1:** The number of putative sulfate transporter proteins present in the complete genomes of 44 SRMs for each transport protein family.

Phylum	Genome	Salinity	T(°C)	Transporter Family					Reference
				SulP	DASS	CysP	CysZ	SulT	
Actinobacteria	*Mycobacterium tuberculosis* H37Rv			^∗^					Zolotarev et al., 2008
Proteobacteria	*Rhodobacter capsulatus* SB1003				^∗^				Gisin et al., 2010
Firmicutes	*Bacillus subtilis*					^∗^			Mansilla and de Mendoza, 2000
Actinobacteria	*Corynebacterium glutamicum*						^∗^		Rückert et al., 2005
Proteobacteria	*Salmonella enterica*							^∗^	Pflugrath and Quiocho, 1985
Crenarchaeota	*Caldivirga maquilingensis* IC-167	F	85	0	0	1 (19)	4 (20)	0	Itoh et al., 1999
Crenarchaeota	*Vulcanisaeta moutnovskia* 768-28	F	80	0	0	1 (20)	7 (22)	0	Gumerov et al., 2011
Euryarchaeota	*Archaeoglobus fulgidus* 7324	M	76	0	0	1 (25)	10 (19)	0	Beeder et al., 1994
Euryarchaeota	*Archaeoglobus fulgidus* VC-16	F	80	0	0	2 (27)	11 (19)	0	Stetter, 1988
Euryarchaeota	*Archaeoglobus profundus* Avl8	M	82	0	0	2 (28)	4 (24)	0	Burggraf et al., 1990
Euryarchaeota	*Archaeoglobus sulfaticallidus* PM70-1	M	75	0	1 (18)	2 (28)	5 (19)	0	Steinsbu et al., 2010
Firmicutes	*Ammonifex degensii* KC4	F	70	0	0	0	6 (22)	0	Huber et al., 1996
Firmicutes	*Desulfosporosinus orientis* Singapore I	F	30	1 (23)	5 (18)	2 (24)	8 (22)	0	Campbell and Postgate, 1965
Firmicutes	*Desulfosporosinus acidiphilus* SJ4	F	35	3 (23)	0	1 (22)	4 (18)	0	Alazard et al., 2010
Firmicutes	*Desulfosporosinus meridiei* S10	F	28	2 (23)	2 (18)	1 (24)	8 (21)	0	Robertson et al., 2001
Firmicutes	*Desulfotomaculum kuznetsovii* 17	F	65	0	5 (19)	2 (23)	10 (21)	0	Nazina et al., 1989
Firmicutes	*Desulfotomaculum gibsoniae* Groll	F	37	0	9 (20)	0	9 (22)	0	Kuever et al., 1993
Firmicutes	*Desulfotomaculum acetoxidans* 5575	F	36	1 (25)	0	1 (22)	5 (20)	0	Widdel and Pfenning, 1977
Firmicutes	*Desulfotomaculum carboxydivorans* CO-1-SRB	F	55	1 (23)	2 (18)	1 (24)	7(21)	0	Parshina et al., 2005
Firmicutes	*Desulfotomaculum ruminis* DL	I	37	1 (24)	2 (17)	1 (24)	9 (22)	0	Coleman, 1960
Firmicutes	*Desulfotomaculum reducens* MI-l	M	37	2 (24)	4 (18)	1 (25)	9 (18)	0	Visser et al., 2016
Firmicutes	*Thermodesulfobium narugense* Na82	F	55	0	0	0	12 (21)	0	Mori et al., 2003
Nitrospirae	*Thermodesulfovibrio yellowstonii*	F	65	0	0	2 (30)	8 (22)	0	Henry et al., 1994
Proteobacteria	*Desulfobulbus propionicus* lpr3	F	39	2 (24)	2 (17)	1 (21)	9 (22)	0	Widdel and Pfenning, 1982
Proteobacteria	*Desulfatibacillum alkenivorans* AK-01	M	28	1 (20)	3 (18)	1 (22)	9 (21)	0	Cravo-Laureau et al., 2004
Proteobacteria	*Desulfobacca acetoxidans* ASRB2	F	37	0	1 (16)	0	9 (21)	0	Oude Elferink et al., 1999
Proteobacteria	*Desulfobacterium autotrophicum* HRM2	M	25	2 (25)	5 (17)	1 (23)	10 (21)	0	Brysch et al., 1987
Proteobacteria	*Desulfobacula toluolica* Tol2	M	28	1 (23)	5 (20)	2 (21)	4 (22)	0	Rabus et al., 1993
Proteobacteria	*Desulfococcus oleovorans* Hxd3	M	28	1 (24)	4 (18)	1 (22)	3 (19)	0	So et al., 2003
Proteobacteria	*Desulfohalobium retbaense* HR100	M	40	0	0	0	3 (22)	0	Ollivier et al., 1991
Proteobacteria	*Desulfomicrobium baculatum* X	F	30	2 (26)	1 (17)	0	10 (22)	0	Rozanova and Nazina, 1976
Proteobacteria	*Desulfomonile tiedjei* DCB-1	F	37	1 (30)	4 (19)	2 (25)	12 (21)	0	DeWeerd et al., 1990
Proteobacteria	*Desulfovibrio gigas*	F	35	1 (27)	2 (24)	1 (25)	6 (23)	0	Boyle et al., 1999
Proteobacteria	*Desulfovibrio* sp ND132	F	32	3 (25)	4 (21)	1 (25)	8 (21)	0	Gilmour et al., 2011
Proteobacteria	*Desulfovibrio vulgaris* RCH1	–	35	3 (27)	0	1 (21)	9 (20)	0	Morais-Silva et al., 2014
Proteobacteria	*Desulfovibrio vulgaris* DP4	M	–	3 (29)	0	1 (21)	9 (20)	0	Morais-Silva et al., 2014
Proteobacteria	*Desulfovibrio alaskensis* G20	M	30	3 (29)	2 (17)	1 (25)	5 (24)	0	Hauser et al., 2011
Proteobacteria	*Desulfovibrio salexigens*	M	30	3 (24)	3 (18)	1 (23)	6 (19)	0	Skyring and Trudinger, 1973
Proteobacteria	*Desulfovibrio vulgaris* Miyazaki F	F	37	2 (27)	1 (20)	1 (21)	10 (22)	0	Barton, 2013
Proteobacteria	*Pseudodesulfovibrio aespoeensis* Aspo-2	F	30	3 (24)	2 (22)	1 (25)	6 (22)	0	Cao et al., 2016
Proteobacteria	*Desulfotalea psychrophila* LSv54	M	10	4 (24)	3 (16)	1 (23)	7 (24)	0	Knoblauch et al., 1999
Proteobacteria	*Desulfovibrio magneticus* RS-1	F	30	1 (25)	3 (20.5)	3 (20)	10 (20)	0	Sakaguchi et al., 1993
Proteobacteria	*Desulfovibrio africanus* Walvis Bay	M	30	1 (25)	4 (19)	1 (23)	11 (23)	0	Campbell et al., 1966
Proteobacteria	*Desulfovibrio desulfuricans desulfuricans*	I	37	1 (21)	2 (20)	1 (22)	8 (21)	0	Kuever et al., 2005
Proteobacteria	*Desulfovibrio vulgaris subsp. vulgaris* Hildenborough	F	35	3 (27)	0	1 (21)	9 (20)	0	Butlin et al., 1949
Proteobacteria	*Desulfovibrio piezophilus* C1TLV30	M	30	3 (24)	6 (20)	1 (24)	6 (22)	0	Khelaifia et al., 2011
Thermodesulfobacteria	*Thermodesulfatator indicus* CIR29812	M	70	0	1 (17)	0	7 (20)	0	Moussard et al., 2004
Thermodesulfobacteria	*Thermodesulfobacterium geofontis* OPF15	F	83	0	0	1 (25)	6 (20)	0	Hamilton-Brehm et al., 2013
Thermodesulfobacteria	*Thermodesulfobacterium commune*	F	70	0	1 (17)	1 (27)	4 (20)	0	Zeikus et al., 1983

*Salinity preference is denoted as F (Freshwater), M (Marine), and I (Intestinal). The temperature (T) values represent the reported optimum temperature of growth. The values in parentheses represent the % identity of the most similar putative transporter homolog for the respective genome compared to functionally characterized sulfate transporters of the same protein family (highlighted in gray).*

On average there were 2, 3, 1, and 8 putative sulfate transporter proteins of the SulP, DASS, CysP, and CysZ family per genome analyzed, respectively (**Table 1**). Pairwise sequence comparisons between putative sulfate transporter proteins and a model protein (of experimentally proven sulfate transport activity) revealed a low level of conservation as the majority of the sequences fell in the “twilight zone” (Rost, 1999) of sequence homology with ≤25% full-length identity (**Table 1**). Members of these transporter families are transmembrane proteins where their overall architecture is determined by their interaction with the cell membrane (Olivella et al., 2013). They usually share similar structures but low sequence similarity since only a few conserved residues are required in order to determine the structure of the transmembrane domains of the protein and thus homologs exhibit a low overall degree of sequence conservation (Olivella et al., 2013).

Transporters of the SulP family were absent from some SRM even though they are considered as one of the two main sulfate transport systems in SRM along with the SulT family of transporters (Rabus et al., 2015). Specifically, SulP type putative transporters were absent from all six archaeal genomes and all three thermodesulfobacterial genomes examined, as well as from two out of twenty three proteobacterial genomes (*Desulfobacca acetoxidans*, *Desulfohalobium retbaense*), two out of 11 Firmicute genomes and from the single Nitrospirae genome examined (**Table 1**). There is a strong correlation (*p* = 6.85e^-10^, chi-square test) between the optimal temperature of growth and the absence/presence of *sul*P genes, as all except from one of the genomes that lacked genes encoding for the SulP family of sulfate transporters are thermophiles with optimum temperatures of growth of 40°C and above. This finding could suggest that the SulP family of transporters most likely arose in the bacterial domain and more specifically in the Proteobacteria phylum. However, since deep prokaryotic phylogenetic relationships are uncertain, we can only speculate based on the assumption that Proteobacteria “arose” after Firmicutes, Nitrospirae, and Thermodesulfobacteria (Hug et al., 2016).

The majority of the archaeal genomes examined also lacked genes encoding transporters of the DASS family (**Table 1**). Putative CysZ family sulfate transporters (TSUP family transporters) were present in all 44 genomes examined (**Table 1**). The genomes of the Firmicutes *Ammonifex degensii* and *Thermodesulfobium narugense* Na82 and the proteobacterium *Desulfohalobium retbaense* lacked genes encoding for transporters from the SulP, DASS, and CysP families and we could only identify genes encoding putative sulfate transporters from the CysZ family in these genomes.

There was no significant correlation between the presence of a particular sulfate transporter family and the salt preference of the isolates as putative Na^+^ (DASS) and H^+^ (CysP/SulP) sulfate symporters were identified in the genomes of both marine and freshwater isolates (**Table 1**). This observation agrees with earlier reports that challenged the concept that marine sulfate reducers use only sodium ions for sulfate uptake while freshwater strains use only protons (Stahlmann et al., 1991; Kreke and Cypionka, 1994). A previous study demonstrated that *Desulfomicrobium baculatum*, a freshwater strain, required sodium ions for the transport of sulfate (Kreke and Cypionka, 1995). Therefore, we propose that the preferential use of Na^+^ or H^+^ as the symported ion is most likely not determined by the general salinity preferences of the cell but rather by the specific sulfate transporter protein family present in that given cell.

### The SulT Family of Transporters

The SulT family of transporters (TCDB 3.A.1.6) couple the transport of sulfate with the hydrolysis of ATP. They have been extensively studied in *E. coli* and *Salmonella* sp. both of which assimilate sulfate. The SulT system consists of the periplasmic sulfate binding protein (Sbp), which interacts with the permease components CysT and CysW, and the ATP-binding subunit CysA (Kertesz, 2001). The sulfate permease is encoded as an operon by the cysPTWA genes, while the sbp gene in several bacteria is located separately on the genome. The periplasmic binding protein determines the specificity of the SulT transporter. SulT permeases could also transport thiosulfate, in which case the periplasmic binding protein Sbp is replaced by CysP, the thiosulfate binding protein (Kertesz, 2001). SulT family sulfate transporters have a high affinity for sulfate; the Sbp protein of *S. typhimurium* has a half saturation constant (K_m_) of 0.1 μM, while a similar protein in *Mycobacterium tuberculosis* has a K_m_ for sulfate uptake of 36 μM (Jacobson and Quiocho, 1988; Wooff et al., 2002).

Several lines of evidence, both theoretical and experimental, suggest that members of the SulT family of transporters are not the primary sulfate transporters in SRM despite the presence of several genes annotated as *sulT* in the SRM genomes. In microorganisms that perform assimilatory sulfate reduction, such as *E. coli*, sulfate transport is primarily driven by ATP-hydrolysis, however, in dissimilatory sulfate reducers the investment of ATP for the transport of sulfate into the cell would most likely not allow for energy conservation since the energetic yield from sulfate reduction is already low. Physiological studies with *Desulfovibrio vulgaris* growing on hydrogen as electron donor determined that the net energy conservation per sulfate reduced equals one ATP (Badziong and Thauer, 1978). Even though the overall reaction results in the production of three ATP molecules per sulfate, the activation of sulfate in the first step of the metabolic pathway requires the equivalent of two ATP molecules (Badziong and Thauer, 1978). If the transport of sulfate would cost one ATP per sulfate molecule, assuming that one ATP is hydrolyzed per transport cycle as previously suggested for other ABC-type transporters, then the use of SulT proteins to transport sulfate across the cell membrane would bring the net energy gain to zero per sulfate reduced (Lu et al., 2005). Therefore it is unlikely that SulT transporters are involved in the transport of sulfate in dissimilatory sulfate reducers, and agrees with the absence of genes encoding SulT transporters in the analyzed SRM genomes (**Table 1**).

The energetic cost of sulfate uptake in SRMs is thus expected to be less than one ATP. Although both low- and high-affinity transport requires the translocation of protons or sodium ions across the cell membrane, the low affinity electroneutral transport will not consume any energy if the end product of sulfate reduction, sulfide, leaves the cell as H_2_S by diffusion. The assumption that sulfide leaves the cell as H_2_S by diffusion and partially dissociates to HS^-^ and H^+^ once outside of the cell is supported by earlier observations in *Desulfovibrio desulfuricans* (Cypionka, 1987). On the contrary, high-affinity transport requires the translocation of three protons per sulfate, which results in an energy loss of 3/4 of an ATP or one ATP if we assume that four or three protons are transported per ATP formed, respectively (Hoehler et al., 2001). This energy loss is partially compensated by the release of sulfide outside of the cell that is equivalent to the release of two protons, which results in the net consumption of 1/4 or 1/3 of an ATP for electrogenic transport.

Further evidence for the absence of ABC-driven sulfate transporters in SRM comes from experiments where ATPase inhibitors were used to inhibit ATP-driven transport. Such treatments had no effect on the transport of sulfate in *Desulfovibrio desulfuricans*, *Desulfobacterium autotrophicum*, and *Desulfovibrio salexigens* suggesting that SulT family proteins are not the primary sulfate transporters in the strains tested (Cypionka, 1989; Stahlmann et al., 1991). Both *Desulfobacterium autotrophicum* and *Desulfovibrio desulfuricans* strains have genes that encode for putative electrogenic sulfate transporters of the SulP, DASS, CysP, and CysZ families (**Table 1**).

Finally, molybdate ABC-type transporters (TCDB 3.A.1.6.8) could potentially facilitate transport of sulfate across the membrane due to structural similarities between the anions of molybdate and sulfate as has been previously reported for the molybdate (ModABC) transport system of *E. coli* (Maupin-Furlow et al., 1995). However, in previous studies where molybdate was added to cell suspensions of *Desulfovibrio desulfuricans* no effect was observed on sulfate transport confirming again that SulT family transporters are unlikely to be involved in the transport of sulfate (Cypionka, 1989). It is worthy to note that both the sulfate (Sbp) and the molybdate (ModB) permeases are targeted by the same PFAM model (PF00528) most likely resulting to the misannotation of molybdate permeases as sulfate permeases in the SRM genomes.

### The SulP Family of Transporters

SulP proteins function as inorganic anion uptake carriers or anion:anion exchange transporters that can transport a wide range of monovalent and divalent anions such as sulfate, chloride, iodine, formate, and bicarbonate (Mount and Romero, 2004). Most of the information about the structure and function of this family of proteins comes from studies in eukaryotes. SulP proteins, irrespective of their substrate specificity, can act as either low or high affinity transporters (Saier et al., 1999). Even though they can operate with either H^+^ or Na^+^ symport, the majority of the SulP sulfate transporters are reported to be operating in symport with protons (Hawkesford, 2003). Proteins of this family are assembled as dimers of two identical subunits and are predicted to have 10–14 TMHs and a C-terminal cytoplasmic STAS (Sulfate Transporter and Anti-Sigma factor antagonist) domain that may be involved in the regulation of the transporter activity via protein:protein interactions (Shibagaki and Grossman, 2006). Studies of eukaryotic SulP sulfate transporters revealed that mutations in the STAS domain resulted in inhibition of sulfate transport (Rouached et al., 2005).

Only a handful of SulP transporters have been characterized in prokaryotes, the majority of which are bicarbonate: Na^+^ symporters (Price et al., 2004; Karinou et al., 2013). The only prokaryotic SulP-type sulfate transporter protein that has been characterized so far is Rv1739c from *M. tuberculosis* (Zolotarev et al., 2008). Expression of Rv1739c in *E. coli* increased sulfate uptake, while its sulfate transport activity was inhibited by thiosulfate, sulfite, and selenite (Zolotarev et al., 2008). The STAS domain of Rv1739c is believed to bind guanine nucleotides as a means of sensing environmental and metabolic stresses in order to modulate anion transport (Sharma et al., 2012).

#### Phylogenetic Analysis

**FIGURE 1:**
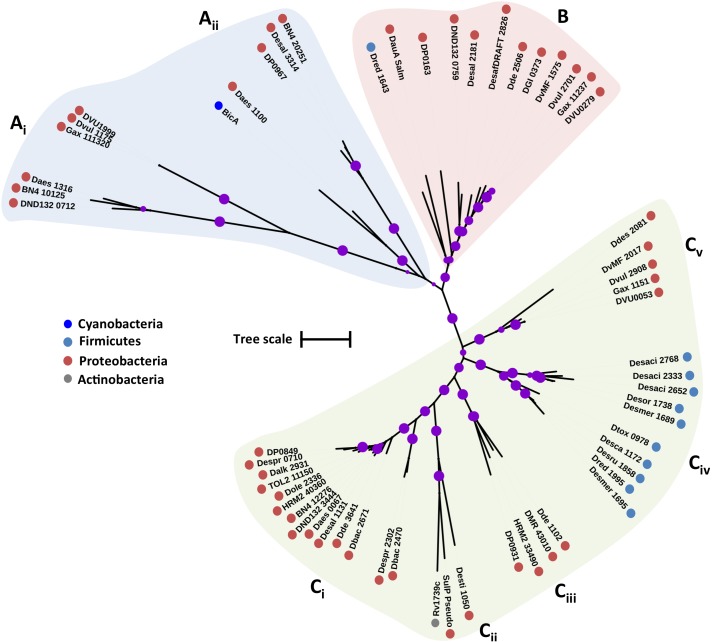
Maximum likelihood phylogenetic tree of SulP protein sequences. The different protein clades are marked with different colored backgrounds and given a letter label **(A–C)**, while separate protein groupings are denoted by subscript numbering (i, ii, etc.). Phylum level classification of the protein sequences is indicated by colored dots next to the accession number (see Supplementary Table 1 for organism information). Reference sequences: BicA, bicarbonate transporter (UniProt: Q14SY0), DauA_Salm, dicarboxylic acid transporter (ACY88617), Rv1739c, sulfate transporter (SulP_Mt), SulP_Pseudo, sulfate transporter (PA1647). Bootstrap values ≥ 50% are indicated by purple dots on each branch where the size of the dot is proportional to the value. The scale bar represents 1 substitution per amino acid position.

The putative SulP family proteins encoded in the examined SRM genomes fall into three major clades (**Figure 1**). Each of the three clades cluster with a different functionally characterized SulP homolog but the majority of the identified SulP protein sequences cluster within the sulfate transporter clade (Clade C) suggesting the presence of several SulP-type sulfate transporter paralogs among SRM (**Figure 1**). Clade A encompasses sequences that cluster with BicA, a low affinity SulP-type bicarbonate:Na^+^ symporter from *Synechococcus* sp. (Price et al., 2004). SulP family proteins that transport bicarbonate ions are frequently misannotated as sulfate transporters (Price et al., 2004). Clade A sequences form two separate groups based on sequence length where group A_i_ contains sequences which are 900–700 amino acids (a. a.) long while group A_ii_ contains sequences of an average length of 540 a. a. and thus, resembles the BicA length (566 a.a.) from *Synechococcus* sp. Clade B includes sequences that cluster with DauA, a SulP-type dicarboxylic acid transporter from *E. coli* (Karinou et al., 2013). Clade C includes sequences that cluster with the SulP-type sulfate transporter Rv1739c from *M. tuberculosis* and a putative SulP-type sulfate transporter from *Pseudomonas aeruginosa* (PA1647) (Tralau et al., 2007; Zolotarev et al., 2008). The model SulP-type sulfate transporters Rv1739c and PA1647 (560 a.a.) form group C_ii_ along with Desti_1050, a 630 amino acid long sequence from *Desulfomonile tiedjei*. Group C_i_ contains putative sulfate transporter sequences from Firmicutes and Proteobacteria with an average length of 710 a.a. due to an insertion of approximately 90 a.a. relative to the model SulP sulfate transport proteins (Rv1739c and PA1647) and results in the presence of an additional transmembrane helix. Group C_iii_ includes sequences from Firmicutes and Proteobacteria with lengths ranging from 590 to 640 a.a while group C_iv_ contains sequences only from Firmicutes and C_v_ only from *Desulfovibrio* sp. (Proteobacteria). We propose that sequences from clades A and B are most likely putative bicarbonate/dicarboxylic acid transporters, while sequences that fall within clade C are more likely to function as sulfate transporters.

#### Sequence Analysis

**FIGURE 2:**
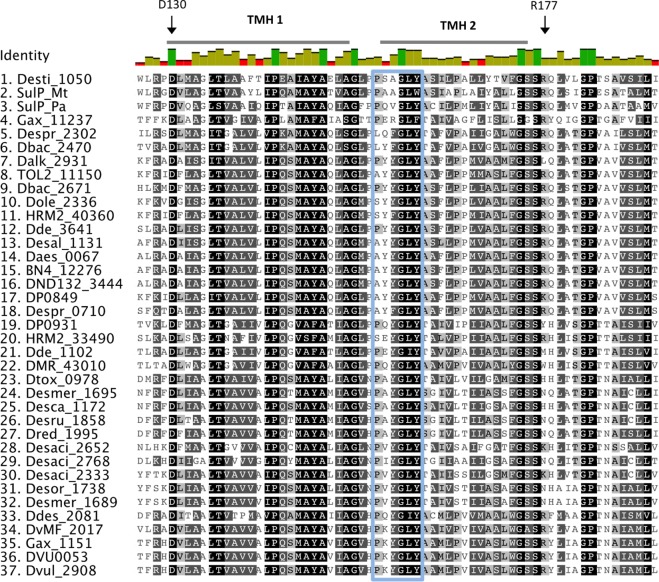
Alignment of putative SulP sulfate transporters. The transporter sequences were selected based on their phylogenetic relationship to the model SulP sequences (**Figure 1**). Invariant, conserved, and non-conserved residues are indicated by green, olive and red bar chart, respectively. Predicted transmembrane helices (TMH) for SulP_Mt (sulfate transporter) are shown by a gray bar above the sequence. Conserved residues are also highlighted within the alignment by gray shading, the darker the shading the higher conservation. Residues of interest are indicated by black arrows, while the PXYGLY motif is highlighted with a blue box.

Sequence alignment of the putative sulfate transporters from clade C showed that there are several conserved residues (**Figure 2**). From studies in eukaryotic SulP-type sulfate transporters it was recognized that the first three transmembrane helices (TMH) have an important role in sulfate transport (Shelden et al., 2001). The first two putative TMHs and the loop between helices 1 and 2 showed high sequence and length conservation suggesting that their conserved residues are important for the function of the protein (Leves et al., 2008). Within those helices there are several charged amino acids that are likely to be important for the function of the protein since the energetic cost associated with the insertion of charged amino acids into the membrane is high (Shelden et al., 2003). Charged amino acids within TMH can be either involved in the transport mechanism or have a structural role (Shelden et al., 2003). The negatively charged aspartic acid residue D130 located at the cytoplasmic border of TMH 1 is conserved in all sequences and was previously demonstrated to be essential for sulfate transport (**Figure 2**) (Loughlin et al., 2002; Shelden et al., 2003). Another important charged residue is arginine R177 that was previously shown to result in loss of up to 80% of sulfate transport activity when mutated (Shelden et al., 2003). R177 is conserved in most SRM sequences except from the majority of the Firmicute sequences where it is mainly replaced by non-charged residues and in two cases replaced by a positively charged lysine (K) (**Figure 2**).

Eukaryotic SulP sulfate transporters are recognized by a highly conserved amino acid motif PXYGLY that forms part of the outside loop and the periplasmic border of TMH2 (Loughlin et al., 2002). The PXYGLY motif is found in the model sulfate transport protein sequences Rv1739c from *M. tuberculosis* and PA1647 from *P. aeruginosa* with different degrees of conservation (**Figure 2**). The PXYGLY motif can also be found in the putative SulP sulfate transporter of clade C where the GLY residues appear to be highly conserved (**Figure 2**). The PXYGLY motif is absent or has a low degree of conservation in the clade A and B sequences which supports our prediction that they most likely do not operate as sulfate transporters (**Figure 1**). The proline residue (P156) that forms part of the motif is less conserved; it is replaced by leucine (L), alanine (A), or serine (S) in certain proteins (**Figure 2**). When substituted with a leucine in the sulfate transporter involved in assimilatory sulfate uptake in the legume, *Stylosanthes hamata*, an almost complete loss of function is observed suggesting that SulP members with this substitution, such as Despr_2302 and Dbac_2470 from *Desulfobulbus propionicus* and *Desulfomicrobium baculatum*, respectively are not functional sulfate transporters (**Figure 2**) (Loughlin et al., 2002). Both Despr_2302 and Dbac_2470 are part of the C_i_ group of longer than usual putative SulP sulfate transporters (**Figure 1**). In the same group the majority of the sequences have either alanine (A) or serine (S) residues at position 156 instead of the conserved proline (P) (**Figure 2**). In previous studies substitution of P156 with alanine (A) resulted in reduced protein trafficking to the membrane, while any transporter protein that was correctly positioned at the membrane exhibited increased affinity for sulfate (Loughlin et al., 2002). We have also identified another SulP-type putative transporter protein in the genome of *Desulfomicrobium baculatum* (Dbac_2671) that has the fully conserved PXYGLY motif (**Figure 2**). The PXYGLY motif is fully conserved in most of the sequences of group C_ii_, and all the sequences found in groups C_iv_ and C_v_ (**Figures 1**, **2**).

Other highly conserved residues with a predicted functional role in the transport of sulfate include Q144 and Y148 from TMH1 and Y161, P167, and Y170 from TMH2 since substitutions of those residues resulted in loss of sulfate transport in the legume, *Stylosanthes hamata* (Leves et al., 2008). Interestingly, Y170 is replaced by alanine (A) in most of the SRM sequences examined, however the importance of such an observation cannot be established in the absence of experimental data (**Figure 2**). In plants the substitution of another conserved residue (D145) to alanine (A) resulted in higher affinity for sulfate (μM) and we can only speculate that such substitution might also have a beneficial effect on the transporter affinity for sulfate (Shelden et al., 2003; Leves et al., 2008).

### The CysP Family of Transporters

CysP is the only characterized sulfate transporter so far within the PiT superfamily of transport proteins and is predicted to operate by sulfate: H^+^ symport. Studies with *E. coli* sulfate-transport mutants expressing the *B. subtilis* CysP gene confirmed that CysP is a sulfate transporter (Mansilla and de Mendoza, 2000). The *B. subtilis* CysP is encoded in an operon that includes genes involved in sulfur metabolism, such as the sulfate adenylyltransferase gene (*sat*) (Aguilar-Barajas et al., 2011). CysP in *B. subtilis* is predicted to have 10–12 TMHs and two homologous domains that most likely resulted from internal gene duplication (Mansilla and de Mendoza, 2000).

#### Phylogenetic Analysis

**FIGURE 3:**
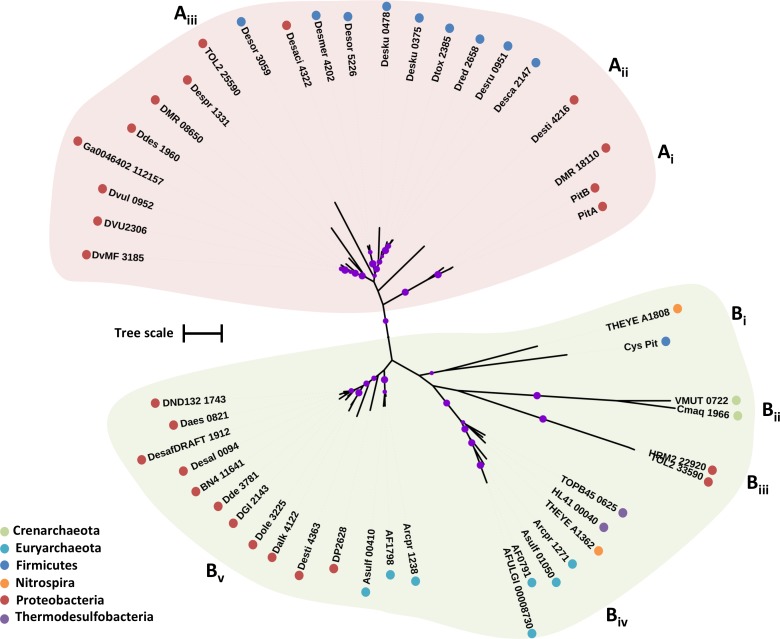
Maximum likelihood phylogenetic tree of CysP protein sequences. The different protein clades are marked with different colored backgrounds and given a letter label **(A,B)**, while separate protein groupings are denoted by subscript numbering (i, ii, etc.). Phylum level classification of the protein sequences is indicated by colored dots next to the accession number (see Supplementary Table 1 for organism information). Reference sequences: PitA, inorganic phosphate transporter (UniProt P0AFJ7), PitB, inorganic phosphate transporter (UniProt P43676), Cys_Pit, sulfate transporter (BSUA_01689). Bootstrap values ≥ 50% are indicated by purple dots on each branch where the size of the dot is proportional to the value. The scale bar represents 1 substitution per amino acid position.

Previous phylogenetic analysis of PiT permeases revealed that CysP is part of a unique cluster of PiT proteins; residues critical for phosphate transport are not conserved in this cluster suggesting that CysP-like PiT permeases are not involved in phosphate transport (Aguilar-Barajas et al., 2011). The putative CysP transport family proteins from the examined SRM fall into two clades; clade A that includes two inorganic phosphate transporters from *E. coli* (PitA and PitB) and clade B that includes the CysP sulfate transporter (Cys_Pit) from *B. subtilis* (**Figure 3**) (Harris et al., 2001). The CysP sulfate transporter forms a group with THEYE_A1808 from *Thermodesulfovibrio yellowstonii* (**Figure 3**). Two archaeal CysP putative transporters form group B_ii_ with shorter sequences (296 a.a.) compared to the CysP from *B. subtilis* (354 a.a.) (**Figure 3**). B_iii_ includes two deltaproteobacterial sequences of 750 a.a. that most likely are not functioning as sulfate transporters (**Figure 3**). The group B_iv_ is formed by sequences that belong to thermophilic isolates with an average length of 310 a.a. (**Figure 3** and **Table 1**). Finally, group B_v_ includes sequences with an average length of 412 a.a. due to an insertion of approximately 60 a.a. in the middle of the protein adding two more predicted TMHs relatively to the sequence of CysP from *B. subtilis*. Within B_v_ the archaeal sequences form a separate sub-group from the proteobacterial sequences suggestive of minimal recent lateral CysP gene transfer between Proteobacteria and Archaea.

#### Sequence Analysis

**FIGURE 4:**
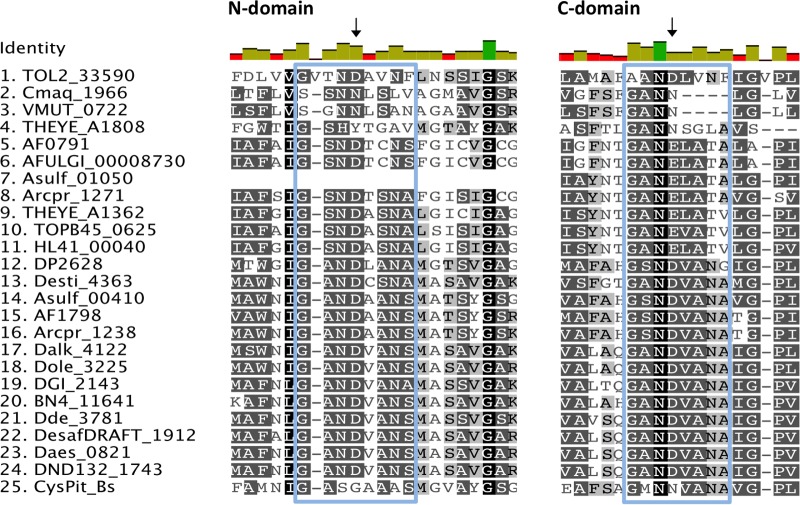
Alignment of selected putative CysP sulfate transporters. The selected transporter sequences clustered with the model CysP sequence on the phylogenetic tree (Clade B, **Figure 3**). Invariant, conserved, and non-conserved residues are indicated by green, olive and red bar charts, respectively. Conserved residues are also highlighted within the alignment by gray shading, the darker the shading the higher conservation. Residues of interest are indicated by black arrows, while the GANDVANA motif is highlighted with a blue box.

The inorganic phosphate transport proteins of the PiT superfamily have at their N- and C-terminal domains a common conserved signature sequence GANDVANA (Bøttger and Pedersen, 2005). These two conserved motifs contain highly conserved aspartate (D) residues essential for phosphate transport that are replaced by glycine (G) and asparagine (N) in the CysP sulfate transporter of *B. subtilis* (Aguilar-Barajas et al., 2011). It is speculated that the substitution of the conserved aspartate residues allows for the substrate specificity of the transport protein for sulfate for CysP instead of inorganic phosphate (Aguilar-Barajas et al., 2011). The GANDVANA motif is also present with variable degrees of conservation in the analyzed SRM sequences. More specifically, 43 out of the 46 sequences have in their N-domain motif the conserved aspartate residue, apart from the archaeal Cmaq_1966 and VMUT_0722 where it is replaced by asparagine (N) and the *Thermodesulfovibrio yellowstonii* THEYE_A1808 sequence where it is replaced by tyrosine (Y) (**Figure 4**). Close examination of the clade B putative sulfate transporter sequences in the examined SRMs reveals that the GANDVANA motif is absent from the N-domain of the archaeal Asulf_01050 sequence (**Figure 4**). At the C-domain the aspartate (D) residue of the GANDVANA motif is conserved for all the sequences within clade A. In clade B sequences we observed various degrees of conservation of the GANDVANA motif. Group B_iii_ and B_v_ sequences have a highly conserved GANDVANA motif (**Figures 3**, **4**). In group B_i_ and B_ii_ sequences the aspartate residue is substituted with asparagine (N) (**Figure 4**). Interestingly, in the sequences forming group B_iv_ the GANDVANA motif is replaced by a GANELAT motif (**Figure 4**). Based on the conservation of the aspartate residue within the GANDVANA motif, it appears that only the CysP proteins found in thermophilic isolates are likely to function as sulfate transporters as the presence of the conserved aspartate residue is indicative of a primarily phosphate transporter function. Further evidence for the importance of the CysP proteins in the analyzed thermophilic isolates comes from the absence of SulP and DASS family transporters from the majority of their genomes (**Table 1**).

### The DASS Family of Transporters

The DASS family includes organic dicarboxylates, inorganic dianions, sulfate, and phosphate transporters (Saier et al., 1999). Proteins of this family are generally divided into the Na^+^-sulfate cotransporters and the Na^+^-carboxylate cotransporters (Markovich and Murer, 2004). Most of the functional and structural information about this family comes from studies in eukaryotic cells. The members of the family that function as sulfate transporters are predicted to have 12 TMHs (Pajor, 2006). Plant members of the DASS family of sulfate transporters function as H^+^-coupled symporters, a possible environmental adaptation to acidic (H^+^-rich) soils (Leustek and Saito, 1999). DASS family members contain in their sequences putative phosphorylation sites that could allow for regulation of the transporter activity (Markovich and Murer, 2004). In eukaryotes NaS genes encode for high affinity electrogenic DASS sulfate transporters whose expression is down-regulated by high sulfate concentrations (Markovich and Murer, 2004). Three Na^+^ ions are coupled to the transport of one sulfate molecule resulting in electrogenic transport and the net transfer of one positive charge across the membrane (Pajor, 2006). A DASS family sulfate transporter (YP_003577054) was identified and characterized in the Alphaproteobacterium *R. capsulatum* (Gisin et al., 2010; Hoffmann et al., 2017). Recently, more DASS family sulfate transporters were characterized in the assimilatory sulfate-reducing Alphaproteobacteria *Rhodobacter sphaeroides*, *Sinorhizobium melioti*, *Agrobacterium tumefaciens*, *Dinoroseobacter shibae* and the gammaproteobacterium *Pseudomonas stutzeri* (Hoffmann et al., 2017). Contrary to the characterized eukaryotic DASS sulfate transporters the prokaryotic homologues exhibited variable transport efficiency and overall they appeared to function as low affinity transporters (Gisin et al., 2010; Hoffmann et al., 2017). Variable sulfate transport affinity by DASS-family putative sulfate transporters was also observed in the sulfate reducer *Desulfobacterium autotrophicum* where the DASS family transporter HRM2_38300 was preferentially expressed in the presence of excess sulfate (mM), while the DASS family transporter HRM2_40290 was preferentially expressed in the presence of less than 100 μM sulfate (Tarpgaard et al., 2017).

#### Phylogenetic Analysis

**FIGURE 5:**
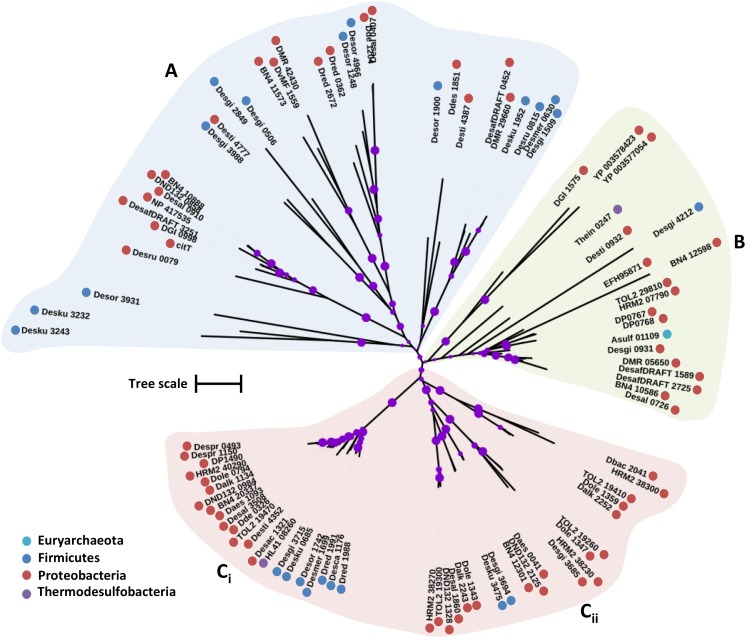
Maximum likelihood phylogenetic tree of DASS protein sequences. The different protein clades are marked with different colored backgrounds and given a letter label **(A–C)**, while separate protein groupings are denoted by subscript numbering (i, ii, etc.). Phylum level classification of the protein sequences is indicated by colored dots next to the accession number (see Supplementary Table 1 for organism information). Reference sequences: citT, citrate/succinate antiporter (UniProt P0AE74), YP_003577054 (DASS sulfate transporter in *R. capsulatus*), EFH95871 (SdcS, sodium-dependent dicarboxylate transporter), NP_417535 (ttdT, L-tartrate/succinate antiporter). Bootstrap values ≥ 50% are indicated by purple dots on each branch where the size of the dot is proportional to the value. The scale bar represents 1 substitution per amino acid position.

Based on phylogenetic analysis, DASS family proteins from the examined SRM could be divided in three major clades (**Figure 5**). Clade A includes proteobacterial and firmicute sequences that cluster with a L-tartrate/succinate antiporter (NP_417535) and a citrate/succinate antiporter (CitT) from *E. coli* (Pos et al., 1998; Kim and Unden, 2007). Clade A sequences have an average length of 460 a.a. with the exception of Desgi2849 which is 1009 a.a. long. Clade B includes highly divergent sequences that cluster with the sulfate transporter (YP_003577054) from *R. capsulatus*. Among the sequences of clade B there is also a dicarboxylate transporter (EFH95871) from *Staphylococcus aureus* hindering any attempts to associate a specific function to the examined sequences based on the phylogenetic analysis alone. The only archaeal DASS transporter sequence identified also falls within the clade B sequences. Clade C is formed by two major groups (**Figure 5**). Group C_i_ consists of highly conserved firmicute, proteobacterial, and thermodesulfobacterial sequences, while group C_ii_ is formed by more divergent sequences with lengths varying from 691 to 474 a.a.

#### Sequence Analysis

Studies with chimeric proteins constructed using various domains from both sulfate and carboxylate transporters of the DASS family revealed that the substrate recognition site of the sulfate transporters is found in the C-terminal domain of the protein (Markovich and Murer, 2004). In eukaryotic cells the DASS sulfate transporters contain serine (S) residues at positions 260 and 288 (TMH 5 and 6), while the carboxylate transporters contain alanine (A) or threonine (T) at those positions suggesting a possible role for those serine (S) residues in sulfate substrate specificity (Li and Pajor, 2003).

**FIGURE 6:**
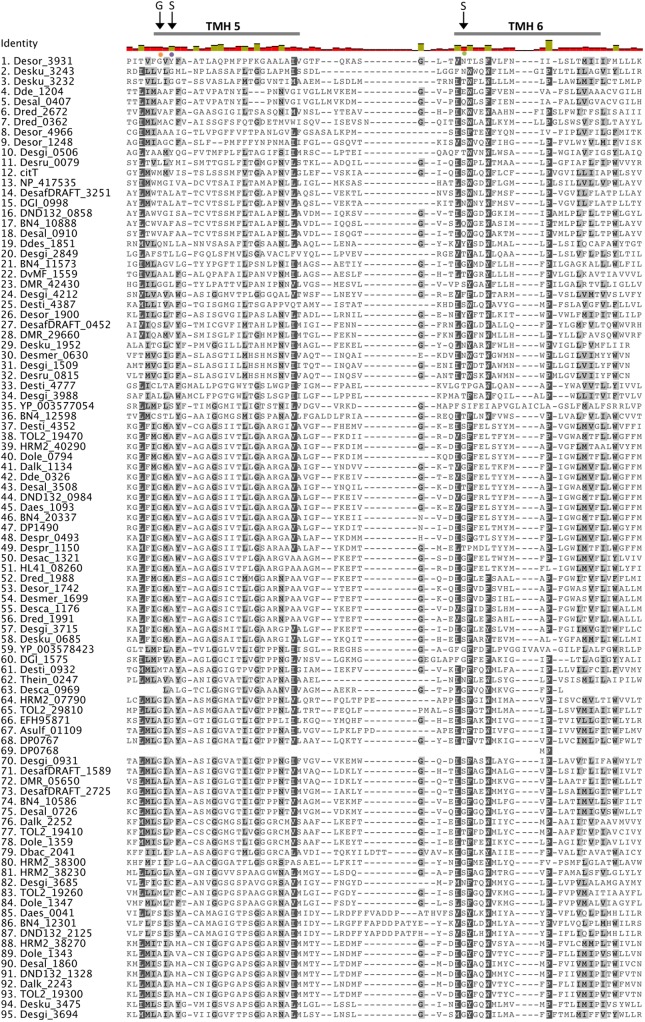
Alignment of putative DASS sulfate transporters. The transporter sequences were selected on their phylogenetic relationship to the model DASS sequences. Invariant, conserved, and non-conserved residues are indicated by green, olive and red bar chart, respectively. Residues of interest are indicated by black arrows.

Alignment of the putative sulfate transporter sequences from the examined genomes revealed that even though the serine residue at position 260 (TMH 5) is conserved in the sulfate transporter (YP_003577054) from *R. capsulatus*, there are only three SRM DASS sequences where the serine residue is also conserved (**Figure 6**). These three sequences belong to *Desulfovibrio* species, Daes_0041 from *Desulfovibrio aespoeensis*, DND132_2125 from *Desulfovibrio sp.* ND132, and BN4_12301 from *Desulfovibrio piezophilus*, and cluster together within group C_ii_ (**Figure 5**). All the sequences from clade A, which clustered with the citrate/succinate antiporter (CitT) from *E. coli*, had substituted S260 with either an amino acid with a hydrophobic side chain (I, V, L, W, F) or a special (G, C) amino acid (**Figure 6**). Similarly the clade A affiliated citrate/succinate antiporter (CitT), the L-tartare/succinate antiporter (NP_417535), and the dicarboxylate transporter (EFH95871) have a valine (V), an isoleucine (I), and a glycine (G), respectively, at position 260 and not alanine or threonine as would have been expected based on previous studies of the eukaryotic DASS family carboxylate transporters (**Figure 6**). The majority of the sequences in clade B and C had an alanine at position 260 with the exception of a fraction of sequences (Dbac_2041, HRM2_38300, TOL2_19410, Dole_1359, Dalk_2252) from group C_ii_ where S260 is substituted by a proline (P) could suggest that in prokaryotic DASS-family sulfate transporters the amino acid at position 260 might not have such a crucial role in determining substrate specificity.

Mutation of the glycine (G) residue at position 258 into a cysteine (C) caused complete loss of sulfate transport activity in eukaryotic sulfate DASS transporters (Li and Pajor, 2003). Even though, G258 is conserved in the majority of the sequences in clade B (TOL2_29810, HRM2_07790, DP0767, DP0768, Asulf_01109, Desgi_0931, DesafDRAFT_1589, DesafDRAFT_2725, BN4_10586, Desal_0726) and all sequences in group C_i_, the sulfate transporter (YP_003577054) from *R. capsulatus* has a proline (P) at position 258 (**Figures 5**, **6**). Similarly, mutation of the asparagine (N) residue at position 262 into a cysteine (C) caused complete loss of sulfate transport activity in eukaryotic sulfate DASS transporters (Li and Pajor, 2003). N262 was not conserved in all of the DASS transporter sequences examined, however in a fraction of sequences of group C_ii_ we found conserved asparagine (N) residues at position 264 (**Figures 5**, **6**). Finally, mutation of the serine (S) residue at position 288 resulted in broader cation selectivity in eukaryotic sulfate transporters (Kahn and Pajor, 1999). S288 is conserved in one third of the examined SRM sequences across the three groups (**Figure 6**). The lack of conservation at residue 288 among the majority of the examined sequences could suggest that the DASS transporters in SRM are able to use cations other than sodium in order to perform their transport activity (**Figure 6**). The overall importance of the different substitutions at positions 258, 260, and 288 will have to be determined experimentally as it is hard to predict their effects on transport specificity/efficiency based only on the limited studies of eukaryotic DASS transporters.

### The CysZ Family of Transporters

A CysZ family sulfate transporter has been identified and characterized in the assimilatory sulfate-reducing actinobacterium, *Corynobacterium glutamicum* (Rückert et al., 2005). The CysZ protein of *C. glutamicum* was characterized as a high affinity sulfate transporter since *cys*Z knockout mutants were unable to grow with less than 5 mM sulfate as the only sulfur source while the growth of the mutant was restored at 30 mM sulfate (Rückert et al., 2005). The *cysZ* gene of *C. glutamicum* is part of an operon encoding for genes involved in the assimilatory sulfate reduction pathway (Rückert et al., 2005). CysZ was also purified and characterized in *E. coli* where it transported sulfate at low sulfate concentrations (0.5 μM) with an apparent Km of 0.72 μM (Zhang et al., 2014). In *E. coli*, CysZ sulfate transport activity was inhibited by the presence of sulfite, which is believed to interact with the CysZ transporter and in turn regulate the influx of sulfate into the cell (Zhang et al., 2014).

**FIGURE 7:**
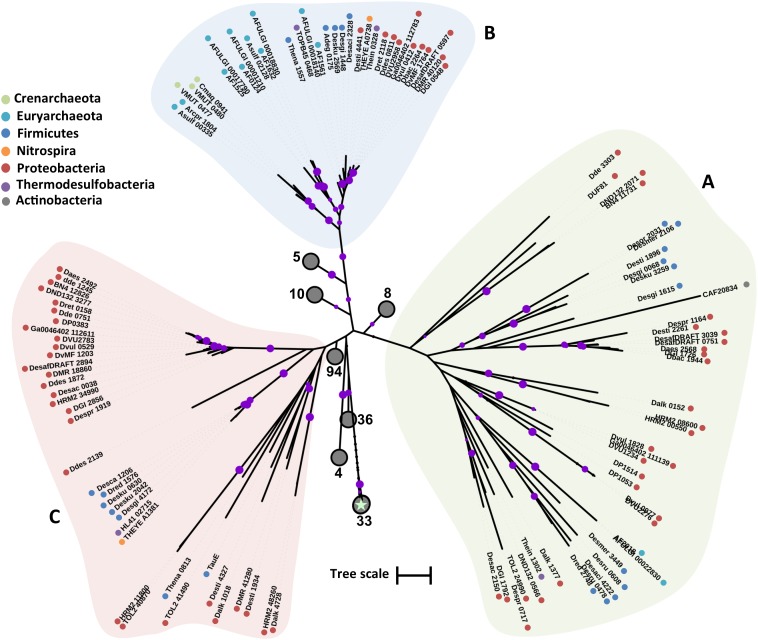
Maximum likelihood phylogenetic tree of CysZ protein sequences. The different protein clades are marked with different colored backgrounds and given a letter label **(A–C)**. Phylum level classification of the protein sequences is indicated by colored dots next to the accession number (see Supplementary Table 1 for organism information). Collapsed nodes are presented as gray circles, the number of collapsed leaves is indicated next to the circle. The *E. coli* CysZ sequence is indicated on the tree by a star. Reference sequences: DUF81, probable sulfite/organosulfonate exporter (UniProt Q0K020), CAF20834 (CysZ sulfate transporter in *C. glutamicum*), TauE, probable sulfite/organosulfonate exporter (UniProt K7WU96). Bootstrap values ≥ 50% are indicated by purple dots on each branch where the size of the dot is proportional to the value. The scale bar represents 1 substitution per amino acid position.

In the absence of appropriate models for the detection of the CysZ sulfate transporters within the TSUP family of proteins, we searched for all TSUP proteins present in the examined genomes. The search identified over 300 proteins of the TSUP superfamily ranging in length from 240 to 790 a.a. Phylogenetic analysis of the TSUP proteins from the examined genomes produced a tree with several clades (**Figure 7**). The characterized CysZ sulfate transporters from *C. glutamicum* (CAF20834) and *E. coli* were located in separate clades of the tree (**Figure 7**). The *E. coli* CysZ sequence appears to be distant from the other TSUP family proteins and clusters within a clade with low bootstrap support. The *C. glutamicum* CysZ protein is also affiliated with a clade with low bootstrap support and is related to a putative sulfite exporter (DUF81) from *Cupriavidus necator*, which is however not related to the functionally characterized sulfite exporter (TauE) from *Oenococcus oeni* (Weinitschke et al., 2007; Favier et al., 2012). TSUP family proteins appear to be abundant in the genomes of SRMs, however in the absence of characterized protein structures and conserved sequence information predictions regarding their putative function are not feasible. In the genomes of *Ammonifex degensii* and *Desulfohalobium retbaense* the only putative sulfate transporter genes found were from the TSUP family (**Table 1**). The putative CysZ sequences from *Ammonifex degensii* (Adeg_0175) and *Desulfohalobium retbaense* (Dret_2118) are part of a highly conserved clade of TSUP sequences with high bootstrap support (**Figure 7**, clade B). The absence of putative sulfate transporter sequences from the DASS, SulP, and CysP protein families in *Ammonifex degensii* and *Desulfohalobium retbaense* suggest that (i) either there are additional protein families that are yet to be characterized as sulfate transporters or (ii) CysZ could potentially act as the primary sulfate transporter in some SRMs. Clade B includes sequences from 25 out of the 44 analyzed SRM genomes across all six phyla (**Figure 7**).

### Genomic Context

**FIGURE 8:**
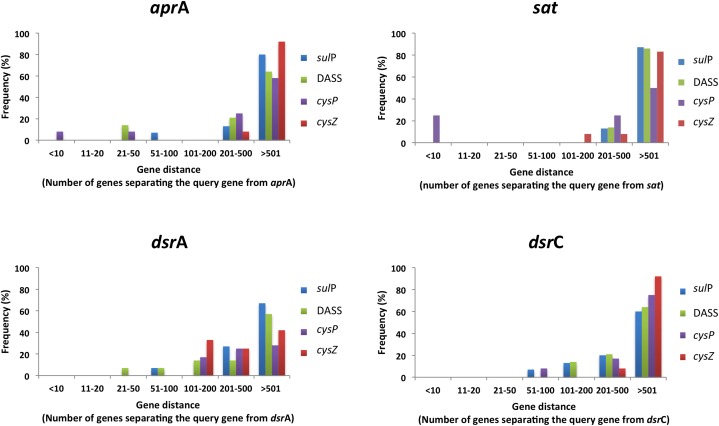
The location of selected putative sulfate transporter genes in relation to genes involved in the sulfate reduction pathway in the examined SRM genomes. Distance was calculated using the locus tag numbers and assumed to correspond to genes away from query. Frequency was calculated as the percentage of putative sulfate tranposrters of a given protein family located a certain number of genes away from the query. The selected putative sulfate genes clustered with the model family protein with at least 50% bootstrap clade support.

The genomic context (location of a particular gene in the genome) of a protein-encoding gene allows for functional associations since often genes that are involved in the same pathway can be found on the same transcriptional unit and/or located closely on the genome (Huynen et al., 2000). For example, CysZ from *C. glutamicum* and CysP from *B. subtilis* are located in operons of genes involved in sulfate assimilation and sulfur metabolism, respectively. We therefore analyzed the genomic context of all putative sulfate transporter encoding genes and calculated their distance from reference genes of the sulfate reduction pathway; sulfate adenylyltransferase (*sat*), adenosine-5′-phosphosulfate reductase alpha subunit (*apr*A), dissimilatory sulfite reductase subunit A (*dsr*A), and dissimilatory sulfite reductase subunit C (*dsr*C). The majority of the identified protein sequences are encoded by genes located 100 or more genes away from genes of the sulfate reduction pathway (Supplementary Figure 1). Putative CysZ transporter encoding genes were found at regular intervals from the reference genes most likely due to the high copy number of TSUP homologs in the examined genomes. In order to evaluate our approach we also reanalyzed a subset of putative sulfate transporter genes based on their phylogenetic groupings by selecting DASS, SulP, CysP, and CysZ proteins that clustered close to the model sulfate transporter protein for each family (**Figure 8**). The majority of the putative sulfate transporter genes were once again located 200 or more genes away from the reference sulfate reduction pathway genes. However, 10–25% of the examined CysP transport genes were located less than 10 genes away from the *sat* gene (**Figure 8**). With the exception of the putative CysP-encoding gene Arcpr_1271 from *Archaeoglobus profundus* that is located 7 genes away from the sat gene, the putative CysP-encoding genes in the thermophilic isolates are located 100s of genes away from the sulfate reduction pathway genes. The majority of the putative sulfate transporter encoding genes in SRM appear to be located randomly in the examined genomes. Whether this is due to the fact that the majority of these genes do not encode for proteins that are involved in sulfate transport or that sulfate transporter genes are not located in close proximity to the genes involved in dissimilatory sulfate reduction remains unclear. Extensive molecular studies will be required in order to understand which of these diverse proteins are involved in sulfate transport and which are not.

**FIGURE 9:**
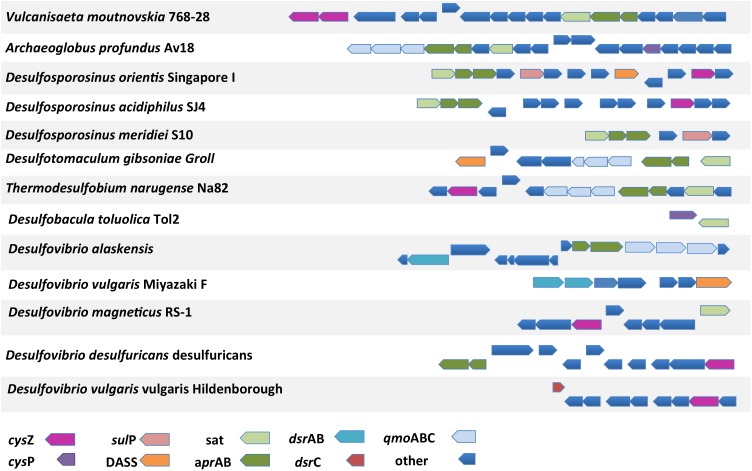
The genomic location of putative sulfate transporter genes and their proximity to genes involved in the sulfate reduction pathway. *sat*, sulfate adenylyltransferase; *apr*A, adenosine-5′-phosphosulfate reductase alpha subunit; *apr*B, adenosine-5′-phosphosulfate reductase beta subunit; *dsr*AB, dissimilatory sulfite reductase alpha or beta subunit; *dsr*C, dissimilatory sulfite reductase C subunit; *qmo*ABC, quinone-modifying oxidoreductase subunit A, B, or C; other, unrelated to sulfate metabolism genes.

**Figure 9** shows all cases from the analyzed SRM genomes where putative sulfate transporter genes are located in close proximity to the reference sulfate reduction pathway genes. Interestingly, all three SulP transporter-encoding genes located in close proximity to genes of the sulfate reduction pathway belong to sequences that cluster within clade C in the SulP phylogenetic tree (**Figures 1**, **9**). Combining the evidence from the phylogenetic and genetic context analysis we propose that Desor_1738 from *Desulfosporosinus orientis*, Desmer_1689 from *Desulfosporosinus meridiei*, and Dde_1102 from *Desulfovibrio alaskensis* are most likely operating as sulfate transporters. Similarly, the CysP encoding transporter genes localized close to the sulfate reduction pathway genes also cluster with the model CysP sulfate transporter sequence in the CysP phylogenetic tree suggesting that Arcpr_1271 from *Archaeoglobus profundus* and TOL2_33590 from *Desulfobacula toluolica* most likely function as sulfate transport proteins (**Figures 3**, **9**). In *Desulfosporosinus orientis* the DASS encoding gene Desor_1742 is located in close proximity to the *sat* and *apr*AB genes as well as a SulP (Desor_1738, see above) and a TSUP (Desor_1745) encoding gene (**Figure 9**). Desgi_3715, a DASS encoding gene from *Desulfomaculum gibsoniae*, is also located in close proximity to the *sat* and *apr*AB genes (**Figure 9**). Desgi_3715 and Desor_1742 are part of the C_i_ group of DASS sequences characterized by the highly conserved G258 residue. Finally, Desaci_2328, a CysZ encoding gene from *Desulfosporosinus acidiphilus*, is located close to the *sat* and *apr*A genes and can be found in clade A sequences on the TSUP phylogenetic tree. The rest of the TSUP encoding genes located in close proximity to genes of the sulfate reduction pathway as shown in **Figure 9**, occur in different clades in the CysZ phylogenetic tree highlighting the high diversity of the TSUP sequences in SRM.

### Combining *in Silico* Predictions With Molecular Studies

The sheer number of putative sulfate transporters identified in the examined genomes and the lack of extensive functional studies of sulfate transporters in prokaryotes (especially in dissimilatory sulfate reducers) renders any predictions regarding the sulfate transport ability of the identified proteins speculative. In this section we will focus on three SRMs and combine our comparative genomics analysis with previously published studies employing laboratory-based approaches.

#### *Desulfovibrio vulgaris* Hildenborough

In the genome of *D. vulgaris* Hildenborough there are three putative SulP type sulfate transporters. DVU0279 that clusters with the dicarboxylic acid transporter DauA (Clade B), DVU1999 that clusters with the bicarbonate transporter BicA (Clade A), and DVU0053 that clusters with the model SulP sulfate transporter protein and is most likely the one that operates as a sulfate transporter (Clade C) (**Figure 1**). Transposon mutation analysis showed that none of the three putative SulP-type transporters in *D. vulgaris* Hildenborough are essential for sulfate transport, suggesting the existence of alternative sulfate transporters (Keller and Wall, 2011). The genome of *D. vulgaris* Hildenborough also encodes for a putative CysP and several CysZ transporter genes that could potentially act as primary sulfate transporters (**Table 1**). DVU2306, the CysP transporter, identified in *D. vulgaris* Hildenborough clusters within clade A that includes two inorganic phosphate transporters from *E. coli* (PitA and PitB) suggesting that it most likely operates as a phosphate transporter (**Figure 3**). Further, evidence suggesting that DVU2306 is not involved in sulfate transport can be drawn from sequence analysis since the highly conserved aspartate (D) residues essential for phosphate transport are conserved in the N- and C-terminal domains of the protein. There are nine genes encoding for TSUP family transporters in the genome of *D. vulgaris* Hildenborough that are phylogenetically diverse and can be found on several different clusters on the CysZ tree (**Figure 7**). Interestingly, DVU2958 clusters within a highly conserved clade of TSUP sequences (clade B) which also includes the only putative sulfate transporters encoded in the genomes of *Ammonifex degensii* and *Desulfohalobium retbaense* (**Figure 7**).

#### *Desulfobacula toluolica* Tol2

The genome of *Desulfobacula toluolica* Tol2 encodes representatives of all four families of sulfate transporters (**Table 1**). Analysis of the membrane-protein fraction confirmed the presence of a SulP (TOL2_16610) and two DASS (TOL2_19410, TOL2_19470) transporters (Wöhlbrand et al., 2016). The SulP2 (TOL2_16610) transporter detected in the proteomic study was not picked up by our search due to low sequence identity (14 %) when compared to the characterized SulP sulfate transporter from *M. tuberculosis*. TOL2_16610 shares higher sequence identity (26 %) with the characterized high affinity molybdate transporter (Mot1) from the eukaryote *Arabidopsis thaliana* (Tomatsu et al., 2007). A query using the relevant PFAM model (PF16983) revealed that SulP-family molybdate transporters can be found in several SRM genomes. Our analysis however identified another SulP type transporter (TOL2_11150) that grouped with the characterized sulfate SulP transporter from *M. tuberculosis* on the SulP phylogenetic tree (**Figure 1**). TOL2_11150 has a sequence identity score of 23% when compared to SulP_Mt. Both DASS transporters detected in the membrane protein fraction are part of the clade C sequences on the DASS tree (**Figure 5**). The two DASS homologues share 27% sequence identity. TOL2_19410 falls within group C_ii_ while TOL2_19470 is found within the C_i_ cluster (**Figure 5**). The presence of two DASS homologues with low sequence identity might be linked to the variable sulfate transport efficiency among different DASS transporters (high vs. low affinity).

#### Desulfobacterium autotrophicum

The genome of *Desulfobacterium autotrophicum* also has genes encoding for all four families of putative sulfate transporters (**Table 1**). Tarpgaard et al. (2017) investigated the transcription levels of the putative DASS and SulP-family transporters in *Desulfobacterium autotrophicum* under low and high sulfate concentrations. At high sulfate concentration (15 mM) the genes encoding a SulP (HRM2_40360) and a DASS (HRM2_40290) family sulfate transporters were highly expressed. HRM2_40360 and HRM2_40290 were also detected in the membrane protein-enriched fractions of *Desulfobacterium autotrophicum* cultures growing in excess of sulfate (Dörries et al., 2016). At low sulfate concentration (<100 μM) genes encoding for two DASS family putative sulfate transporters (HRM2_38230, HRM2_38300) were highly expressed (Tarpgaard et al., 2017). There was no significant change in the expression of another two DASS (HRM2_07790, HRM2_38270) and one SulP (HRM2_33490) family transporters between low and high sulfate grown cells (Tarpgaard et al., 2017). No expression was detected for the SulP encoding gene HRM2_13280 (Tarpgaard et al., 2017). HRM2_13280 shares low sequence identity (14 %) to the characterized SulP sulfate transporter from *M. tuberculosis* and higher sequence identity (28 %) with the characterized high affinity molybdate transporter (Mot1). Thus, HRM2_13280 most likely functions as a molybdate transporter like the aforementioned *Desulfobacula toluolica* SulP-homolog TOL2_16610 with which it shares 64% sequence identity.

Both putative SulP family sulfate transporters, HRM2_33490 and HRM2_40360, were part of the Clade C sequences along with the characterized SulP sulfate transport proteins (**Figure 1**). The characteristic PXYGLY motif found in the sulfate transporters of the SulP-family can be identified with various degrees of conservation in the identified *Desulfobacterium autotrophicum* SulP-family transporters, with HRM2_33490 showing the highest degree of conservation (**Figure 2**). Among the five putative DASS sulfate transporters there was very low sequence conservation with pairwise identity values ranging from 18 to 28%. The high affinity sulfate transporters HRM2_38230 and HRM2_38300 can be found in the C_ii_ group on the DASS tree along with HRM2_38270 whose expression was not affected by changes in the sulfate concentration (**Figure 5**). The low affinity HRM2_40290 transporter can be found among the C_i_ group sequences (**Figure 5**). Could it be that proteins from C_i_ and C_ii_ clusters exhibit different affinities for sulfate? Further molecular studies are required in order to understand which conserved residues are involved in the molecular mechanism of sulfate transport by the different protein families and which residues are responsible for the existence of DASS sulfate transporters with different transport affinities.

## Conclusion

Even though dissimilatory sulfate reduction is a key process in anoxic environments ranging from the deep subseafloor to the human digestive tract, there are still significant gaps in our knowledge of the steps involved in its metabolic pathway. The first step of the pathway is the transport of sulfate across the cell membrane. Our study used comparative genomics to identify the proteins involved in the transport of sulfate in sulfate-reducing Bacteria and Archaea. We identified putative sulfate transporter encoding genes from four transport families and, surprisingly, we found no evidence for the presence of ABC-type sulfate transporters in SRM despite earlier reports (Rabus et al., 2015). SRM have putative sulfate transporters from the SulP, DASS, CysP, and CysZ protein families. SulP transporters are absent from the genomes of thermophilic SRM across bacteria and archaea and we propose that CysP family transporters are the main sulfate transporters in thermophilic SRM. Putative CysZ family sulfate transporters are present in all SRM genomes examined suggesting that they are involved in the transport of sulfate in SRM. We finally discussed the presence of conserved amino acid substitutions in the protein sequences of the sulfate transporters that could be involved in determining substrate specificity and modulate the transporter affinity for sulfate. We have created a roadmap of the sulfate transporter potential in SRM that we hope will inspire further molecular studies in order to understand this key step in the microbial metabolism of sulfate.

## Author Contributions

AM prepared the manuscript under the supervision of KK, HR, and BBJ. All authors commented on the manuscript.

## Conflict of Interest Statement

The authors declare that the research was conducted in the absence of any commercial or financial relationships that could be construed as a potential conflict of interest.
