# Supplementary material for: Sulfate Transporters in Dissimilatory Sulfate Reducing Microorganisms: A Comparative Genomics Analysis
Source: Front Microbiol. 2018 Mar 2;9:309. doi: 10.3389/fmicb.2018.00309 (PMC5840216; doi:10.3389/fmicb.2018.00309)
Supplement: Supplementary file 2 [file Image_1.PDF]

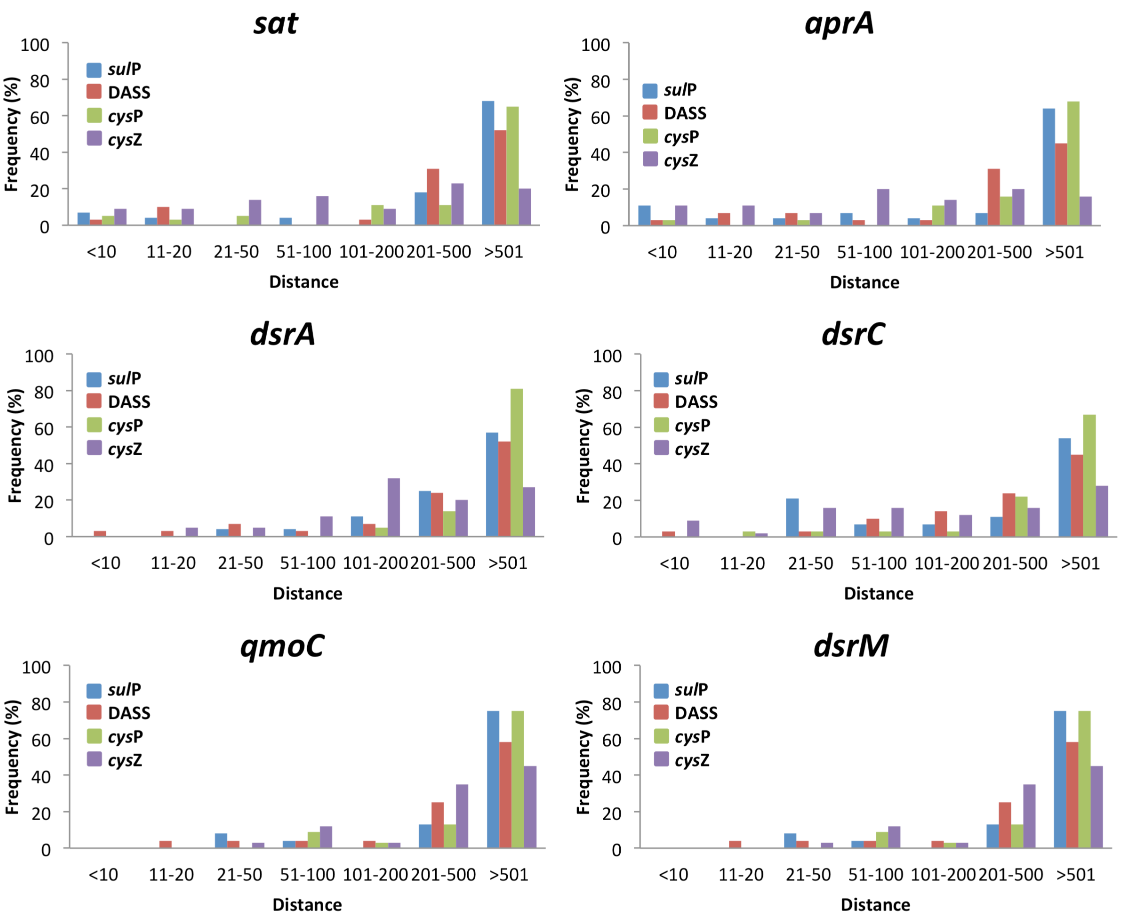

Supplementary Figure 1. The location of putative sulfate transporter genes in relation to genes involved in the sulfate reduction pathway in the examined SRMs. Distance was calculated using the locus tag numbers and assumed to correspond to genes away from query.
